# Expression of interleukin-17RC protein in normal human tissues

**DOI:** 10.1186/1755-7682-1-19

**Published:** 2008-10-17

**Authors:** Dongxia Ge, Zongbing You

**Affiliations:** 1 Department of Structural and Cellular Biology, 1430 Tulane Avenue SL-49, New Orleans, Louisiana 70112, USA; 2 Institute of Biomedical Engineering, West China Medical Center, Sichuan University, No. 3-17 Ren Min Nan Lu Road, Chengdu, Sichuan, 610041, PR China; 3 Tulane Cancer Center, Louisiana Cancer Research Consortium, 1430 Tulane Avenue SL-49, New Orleans, Louisiana 70112, USA; 4 Tulane Center for Aging, Tulane University School of Medicine, 1430 Tulane Avenue SL-49, New Orleans, Louisiana 70112, USA

## Abstract

**Background:**

Interleukin-17 (IL-17) cytokines and receptors play an important role in many autoimmune and inflammatory diseases. IL-17 receptors IL-17RA and IL-17RC have been found to form a heterodimer for mediating the signals of IL-17A and IL-17F cytokines. While the function and signaling pathway of IL-17RA has been revealed, IL-17RC has not been well characterized. The function and signaling pathway of IL-17RC remain largely unknown. The purpose of the present study was to systematically examine IL-17RC protein expression in 53 human tissues.

**Results:**

IL-17RC expression in 51 normal human tissues and two benign tumors (i.e., lymphangioma and parathyroid adenoma) on the tissue microarrays was determined by immunohistochemical staining, using two polyclonal antibodies against IL-17RC. IL-17RC protein was expressed in many cell types including the myocardial cells, vascular and lymphatic endothelial cells, glandular cells (of the adrenal, parathyroid, pituitary, thyroid, pancreas, parotid salivary, and subepidermal glands), epithelial cells (of the esophagus, stomach, intestine, anus, renal tubule, breast, cervix, Fallopian tube, epididymis, seminal vesicle, prostate, gallbladder, bronchus, lung, and skin), oocytes in the ovary, Sertoli cells in the testis, motor neurons in the spinal cord, autonomic ganglia and nerves in the intestine, skeletal muscle cells, adipocytes, articular chondrocytes, and synovial cells. High levels of IL-17RC protein expression were observed in most vascular and lymphatic endothelium and squamous epithelium. The epithelium of the breast, cervix, Fallopian tube, kidney, bladder and bronchus also expressed high levels of IL-17RC, so did the glandular cells in the adrenal cortex, parotid salivary and subepidermal glands. In contrast, IL-17RC protein was not detectable in the smooth muscle cells, fibroblasts, antral mucosa of the stomach, mucosa of the colon, endometrium of the uterus, neurons of the brain, hepatocytes, or lymphocytes. Nevertheless, IL-17RC protein was expressed in the vascular endothelium within the tissues where the IL-17RC-negative cells resided.

**Conclusion:**

IL-17RC protein is expressed in most human tissues, the function of which warrants further investigation.

## Background

Interleukin-17 (IL-17) family has six cytokines, i.e., IL-17A (or IL-17), IL-17B, IL-17C, IL-17D, IL-17E, and IL-17F [1,2]. IL-17A/F heterodimer has also been reported [3,4]. There are five receptors: IL-17RA (or IL-17R), IL-17RB (or IL-17Rh1), IL-17RC (or IL-17RL), IL-17RD, and IL-17RE. IL-17RA is the receptor for IL-17A and IL-17F [5,6]. IL-17RB is the receptor for IL-17B and IL-17E [7,8]. IL-17RD binds to fibroblast growth factor (FGF) receptor and inhibits the FGF receptor-mediated extracellular signal-regulated kinase (ERK) pathway [9-12]. The receptors for IL-17C and IL-17D cytokines have not been identified, nor the cytokines for IL-17RD and IL-17RE. In general, the IL-17 family plays proinflammatory functions [2,13-15]. IL-17A and IL-17F are secreted by a subtype of CD4^+ ^T cells, which are named T helper 17 (T_H_17) [16,17]. IL-17A is also produced by some CD8^+ ^T cells and the T cells expressing αβ or γδ T cell receptor [18-20]. IL-17A and IL-17F contribute to a number of autoimmune and inflammatory diseases such as rheumatoid arthritis [21,22], inflammatory bowel diseases [23,24], multiple sclerosis [17,25,26], organ allograft rejection [27,28], psoriasis [29,30], airway inflammation [31,32], and tumor growth [33,34].

The *IL-17RC *gene located on 3p25.3 has 19 exons. With 22% identity to IL-17RA, the full-length IL-17RC protein is a 720-amino acid type I transmembrane protein with a 20-amino acid signal peptide, a 447-amino acid N-terminal extracellular domain, a 21-amino acid hydrophobic α-helical transmembrane domain, and a 232-amino acid intracellular domain. IL-17RC mRNAs are detected in human prostate, kidney, cartilage, liver, heart, skeletal muscle, and at lower levels in the intestines, brain, lung and spleen [35]. IL-17RC expression was also found in human umbilical vein endothelial cells and chondrocytes and mouse cardiac fibroblasts [36-38]. There are at least 13 mRNA splice isoforms including the full-length and exon(s)-deleted isoforms [39]. The significance of splice variation is unknown. It has been shown that the full-length IL-17RC formed homodimer and inhibited TNFα-induced apoptosis in prostate cancer cells [40]. Homodimerization of IL-17RA has also been reported [41]. IL-17RC forms heterodimers with IL-17RA to mediate IL-17A and IL-17F signals in mouse stromal cells [42] and human gastric adenocarcinoma AGS cells and synoviocytes [43,44]. Recently, it has been shown that IL-17RC functions as a receptor for both IL-17A and IL-17F [45]. Deletion of exon 7 of human IL-17RC does not affect its binding to human IL-17A and IL-17F, but deletion of exon 12 does abolish ligand binding capacity [45]. Interestingly, mouse IL-17A does not bind to any forms of human or mouse IL-17RC, whereas mouse IL-17F only binds to the full-length human and mouse IL-17RC but not to exon-deleted isoforms [45].

We have recently reported that different IL-17RC protein forms were differentially expressed in the immortalized normal prostatic epithelium and prostate cancer cells [46]. Here we report the results of immunohistochemical staining for IL-17RC protein expression in over 50 human normal tissues.

## Results and discussion

In this study, we examined IL-17RC expression in 51 normal human tissues and two benign tumors (i.e., lymphangioma and parathyroid adenoma, due to the difficulties in sampling these normal tissues according to the tissue microarray provider) by immunohistochemical staining, using two different polyclonal antibodies against IL-17RC that have been characterized previously [35,39,40,46,47]. The rabbit anti-IL-17RC intracellular domain antibodies (anti-ICD) that recognize an intracellular domain (peptide sequence: DSYFHPPGTPAPGR) of IL-17RC protein were affinity-purified [35]. The goat anti-IL-17RC extracellular domain antibodies (anti-ECD) were generated using the extracellular domain of human IL-17RC isoform #3 (in which exon 7 was spliced out) as immunogen. Therefore, it is possible that the anti-ECD antibodies may recognize a soluble receptor that contains only the extracellular domain, whereas this soluble receptor can not be recognized by the anti-ICD antibodies. Previously, we have reported that the IL-17RC protein recognized by the anti-ECD antibodies was highly expressed in the normal prostatic epithelium, but significantly decreased or was absent in the prostate cancer cells [46]. In contrast, the IL-17RC protein recognized by the anti-ICD antibodies was expressed at higher levels in the prostate cancer cells than in the normal prostatic epithelium [46]. Both antibodies recognized the same recombinant full-length IL-17RC expressed in 293 cells, although the anti-ECD antibodies had lower affinity than the anti-ICD antibodies in the Western blot analysis. Nevertheless, both antibodies detected similar levels of IL-17RC protein expression in vascular endothelium by immunohistochemical staining [46]. The results of the current study were summarized in Table 1 and described below.

**Table 1:** Expression of IL-17RC protein in human tissues.

**Figure number**	**Age of donor**	**Organ/tissue**	**Cell type**	**Anti-ICD**	**Anti-ECD**
1A/A'	27	Aorta	Smooth muscle	-	-
1B/B'	59	Heart	Myocardium	+	+
1C/C'	27	Lymphangioma	Lymphatic endothelium	+	+++
1D/D'	56	Lung	Small artery, endothelium	+++	+++
1E/E'	49	Intestine	Small vein, endothelium	+++	++
2A/A'	Unknown	Adrenal gland	Cortex, glandular cell	+++	++
2B/B'	Unknown	Adrenal gland	Medulla, glandular cell	+	+
2C/C'	59	Parathyroid	Adenoma, glandular cell	-	++
2D/D'	78	Pituitary	Anterior, glandular cell	+	++
2E/E'	48	Thyroid gland	Glandular cell	+	+
3A/A'	81	Esophagus	Squamous cell	-	+++
3B/B'	43	Stomach	Antral mucosa, epithelium	-	-
3C/C'	53	Stomach	Oxyntic mucosa, epithelium	+	++
3D/D'	45	Small intestine	Mucosa, epithelium	-	+
3E/E'	59	Colon	Mucosa, epithelium	-	-
3F/F'	59	Anus	Mucosa, squamous cell	-	+++
4A/A'	46	Breast	Gland, epithelium	-	+++
4B/B'	55	Cervix	Ectocervix, squamous cell	-	+++
4C/C'	60	Cervix	Endocervix, epithelium	-	++
4D/D'	49	Uterus	Endometrium, secretory	-	-
4E/E'	52	Fallopian tube	epithelium	-	+++
4F/F'	18	Ovary	oocyte	+	+
5A/A'	72	Testis	Seminiferous tubule	++	+
5B/B'	83	Epididymis	epithelium	++	++
5C/C'	56	Seminal vesicle	epithelium	+	+
5D/D'	41	Prostate	epithelium	-	+++
6A/A'	78	Brain	Cerebral cortex, neuron	-	-
6B/B'	78	Brain	Cerebellar cortex, purkinje/granular cell	-	-
6C/C'	78	Brain	Ependymal cell	-	-
6D/D'	78	Hippocampus	Neuron	-	-
6E/E'	78	Spinal cord	Motor neuron	+	+
6F/F'	52	Intestine	Autonomic ganglia	+	+
7A/A'	58	Liver	Hepatocyte	-	-
7B/B'	69	Gallbladder	Epithelium	+	++
7C/C'	43	Pancreas	Glandular cell	-	++
7D/D'	65	Salivary gland, parotid	Glandular cell	+++	+++
8A/A'	62	Kidney	Cortex, glomerulus	+++	+++
8B/B'	91	Kidney	Medulla, epithelium	+	+++
8C/C'	41	Bladder	Transitional epithelium	+	+++
8D/D'	73	Lung	Alveoli, epithelium	+	++
8E/E'	73	Bronchus	Epithelium	-	+++
9A/A'	60	Skin	Squamous cell	-	++
9B/B'	60	Subepidermis	Glandular cell	+	+++
9C/C'	76	Lymph node	Lymphatic cell	-	-
9D/D'	52	Spleen	Lymphatic cell	-	-
9E/E'	32	Thymus	Lymphatic cell	-	-
9F/F'	4	Tonsil	Lymphatic cell	-	-
10A/A'	49	Skeletal muscle	Skeletal muscle cell	-	+
10B/B'	52	Instestine	Smooth muscle cell	-	-
10C/C'	55	Uterus	Smooth muscle cell	-	-
10D/D'	39	Breast	Adipocyte	+	+
10E/E'	62	Cartilage, articular	Chondrocyte	+	-
10F/F'	62	Synovium	Synovial cell	++	-

The intensity of IL-17RC staining for a particular cell type in the tissues was graded in this way: - = no staining; + = weak; ++ = intermediate; +++ = strong. The vascular endothelium had intermediate to strong staining in most tissues.

### Cardiovascular system

**Figure 1:**
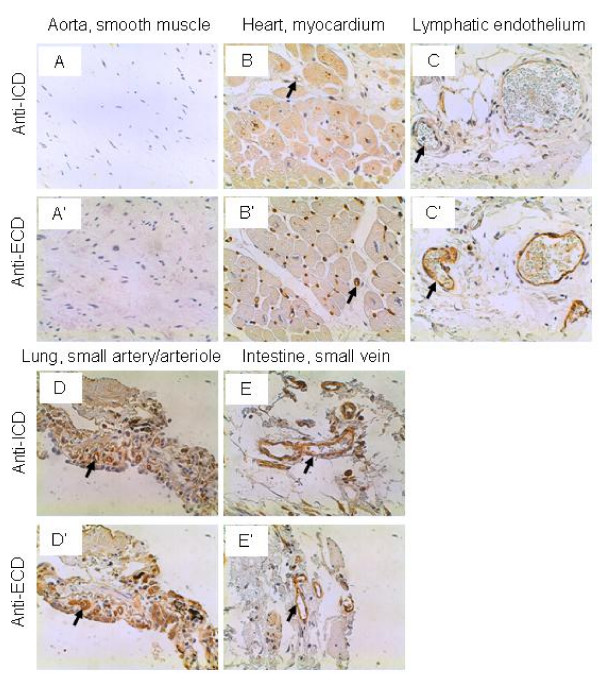
**IL-17RC expression in the cardiovascular system.** Arrows: endothelium. Original magnification: × 400. Figure D, D', E, and E' were reproduced from a previous publication in Neoplasia, 9 (6): 464–470, 2007, with permission by Neoplasia Press and the author Z. You.

The smooth muscle cells in the aorta did not express any IL-17RC as detected by either the anti-ICD or anti-ECD antibodies (Figure 1,A and 1,A'). The myocardial cells had weak staining of IL-17RC by both antibodies, whereas the cardiac vascular endothelium had intermediate to strong IL-17RC staining (Figure 1,B and 1,B'). The lymphatic endothelium in a lymphangioma was stained weakly by the anti-ICD antibodies but strongly by the anti-ECD antibodies (Figure 1,C and 1,C'). The endothelium of the small arteries or arterioles in the lung was stained strongly by both the anti-ICD and anti-ECD antibodies (Figure 1,D and 1,D'). The endothelium of the small veins in the intestine was stained strongly by the anti-ICD anti-bodies and intermediately by the anti-ECD antibodies (Figure 1,E and 1,E'). A previous study suggested that the anti-ICD and anti-ECD antibodies recognize different IL-17RC protein forms [46]. The current results indicate that these different IL-17RC proteins are expressed at similar levels in the vascular and lymphatic endothelium. It has been reported that a combination of hepatocyte growth factor (HGF) and vascular endothelial cell growth factor (VEGF) markedly induced a number of chemokine and cytokines and their receptors (IL-8, IL-6, IL-11, CCR6, CXCR1, CXC1, and IL-17RC) in human umbilical vein endothelial cells [37]. It is unknown what role IL-17RC plays in the endothelium under inflammation.

### Endocrine system

**Figure 2:**
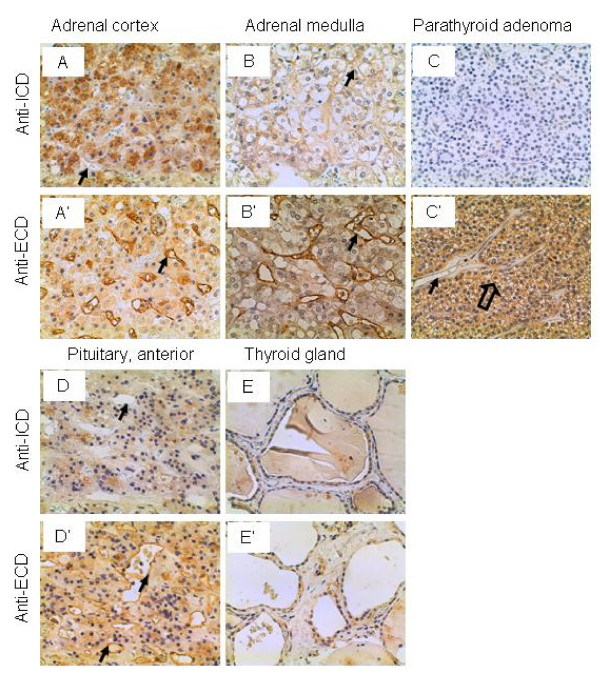
**IL-17RC expression in the endocrine system.** Arrows: endothelium; and open arrow: glandular cells. Original magnification: × 400.

The glandular cells in the adrenal cortex were stained strongly and intermediately by the anti-ICD and anti-ECD antibodies, respectively (Figure 2,A and 2,A'), whereas the glandular cells in the adrenal medulla were stained weakly by both antibodies (Figure 2,B and 2,B'). Interestingly, the vascular endothelium was not stained by the anti-ICD antibodies but stained strongly by the anti-ECD antibodies in both the adrenal cortex and medulla (Figure 2,A/A' and 2B/B'). In a parathyroid adenoma tissue, the glandular cells were not stained by the anti-ICD antibodies but stained intermediately by the anti-ECD antibodies, while the vascular endothelium was not stained by either antibodies (Figure 1,C and 1,C'). The glandular cells of the anterior pituitary gland were stained weakly and intermediately by the anti-ICD and anti-ECD antibodies, respectively, while the vascular endothelium was stained intermediately only by the anti-ECD antibodies (Figure 2,D and 2,D'). The glandular cells of the thyroid gland were stained weakly by both antibodies (Figure 2,E and 2,E'). The function of IL-17RC in the endocrine glands is not known.

### Gastrointestinal tract

**Figure 3:**
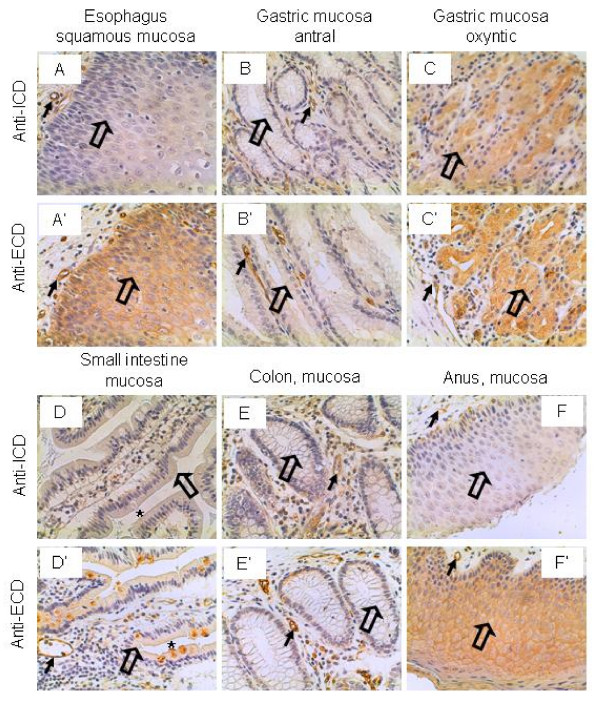
**IL-17RC expression in the gastrointestinal tract.** Arrows: endothelium; open arrows: squamous epithelium (A, A', F, and F') and epithelium (B, B', C, C', D, D', E, and E'); and asterisks: goblet cells (D and D'). Original magnification: × 400.

The esophageal squamous mucosa was not stained by the anti-ICD antibodies but stained strongly by the anti-ECD antibodies, although the vascular endothelium was stained strongly by both antibodies (Figure 3,A and 3,A'). The antral mucosa of the stomach was not stained by either antibodies (Figure 3,B and 3,B'), whereas the oxyntic mucosa was stained weakly and intermediately by the anti-ICD and anti-ECD antibodies, respectively (Figure 3,C and 3,C'). The mucosa of the small intestine was not stained by the anti-ICD antibodies, but stained weakly by the anti-ECD antibodies (Figure 3,D and 3,D'). Interestingly, the goblet cells were stained strongly by the anti-ECD antibodies but not stained by the anti-ICD antibodies (Figure 3,D and 3,D'). The mucosa of the colon was not stained by either antibodies, although the vascular endothelium in the background was stained positively (Figure 3,E and 3,E'). Like in the esophagus, the squamous mucosa of the anus was not stained by the anti-ICD antibodies but stained strongly by the anti-ECD antibodies, although the vascular endothelium was stained positively by both antibodies (Figure 3,F and 3,F'). The significance of differential expression of different IL-17RC proteins in the esophagus and anus is yet to be determined. Given the purported role of IL-17 cytokines in inflammatory bowel diseases [23,24], the absence of IL-17RC in the colon epithelium and its presence in the vascular endothelium suggest that the IL-17 cytokines may act on the vascular epithelium in the lesion to contribute to the local inflammation.

### Breast and female reproductive system

**Figure 4:**
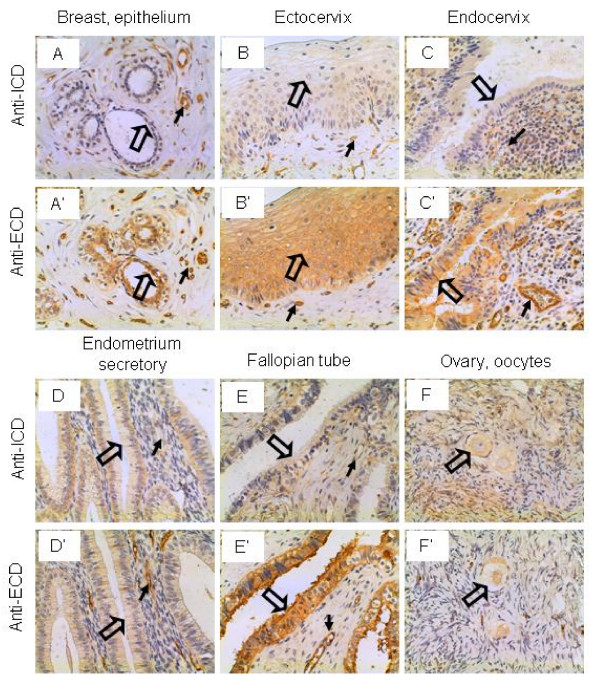
**IL-17RC expression in the breast and female reproductive system.** Arrows: endothelium; open arrows: epithelium (A, A', C, C', D, D', E, and E'), squamous epithelium (B and B'); and oocytes (F and F'). Original magnification: × 400. Figure A and A' were reproduced from a previous publication in Neoplasia, 9 (6): 464–470, 2007, with permission by Neoplasia Press and the author Z. You.

The glandular epithelium of female breast was not stained by the anti-ICD antibodies but stained strongly by the anti-ECD antibodies, although the vascular endothelium was stained strongly by both antibodies (Figure 4,A and 4,A'). It remains to be tested if breast cancer expresses higher levels of IL-17RC that is recognized by the anti-ICD antibodies, as this trend was observed in the prostate [46]. The squamous mucosa of the ectocervix was not stained by the anti-ICD antibodies but stained strongly by the anti-ECD antibodies (Figure 4,B and 4,B'). Similarly, the epithelium of the endocervix was not stained by the anti-ICD antibodies but stained intermediately by the anti-ECD antibodies (Figure 4,C and 4,C'). The secretory endometrium of uterus was not stained by either antibodies (Figure 4,D and 4,D'). In the Fallopian tube, the epithelium was not stained by the anti-ICD antibodies but stained strongly by the anti-ECD antibodies (Figure 4,E and 4,E'). The oocytes in the ovary were stained weakly by both antibodies (Figure 4,F and 4,F'). The function of IL-17RC in these tissues is not known.

### Male reproductive system

**Figure 5:**
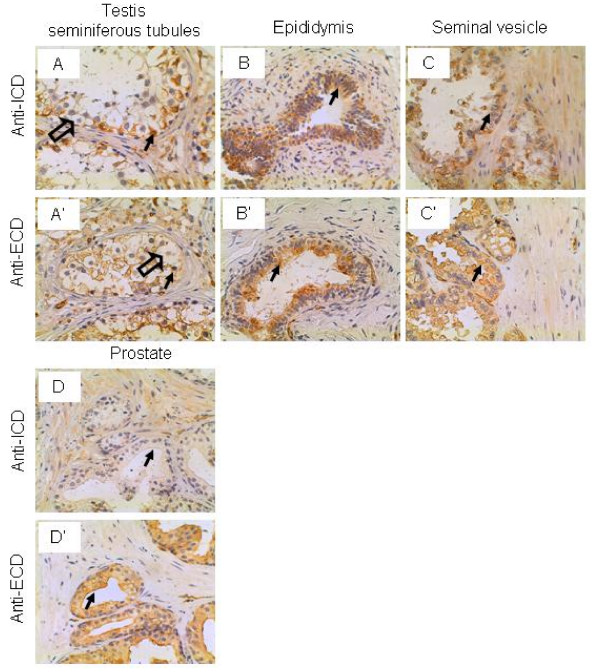
**IL-17RC expression in the male reproductive system.** Arrows: Sertoli cells (A and A') and epithelium (B, B', C, C', D, and D'); and open arrows: spermatogonia (A and A'). Original magnification: × 400. Figure D and D' were reproduced from a previous publication in Neoplasia, 9 (6): 464–470, 2007, with permission by Neoplasia Press and the author Z. You.

In the seminiferous tubule of the testis, the Sertoli cells were stained intermediately and weakly by the anti-ICD and anti-ECD antibodies, respectively, whereas the spermatogonia and spermatocytes were not stained by either antibodies (Figure 5,A and 5,A'). The epithelium of the epididymis and seminal vesicle were stained intermediately and weakly by both antibodies, respectively (Figure 5,B,B' and 5C,C'). In the prostate, the epithelium was not stained by the anti-ICD antibodies but stained strongly by the anti-ECD antibodies (Figure 5,D and 5,D'). We have previously demonstrated that the IL-17RC protein recognized by the anti-ICD antibodies was increased in the prostate cancer cells, whereas the IL-17RC protein recognized by the anti-ECD antibodies was decreased in the prostate cancer cells [46]. We speculate that expression of different IL-17RC proteins may be related to the responsiveness of the epithelial cells to the IL-17 cytokines, which needs to be investigated. The function of IL-17RC in the testis, epididymis and seminal vesicle is not known.

### Nervous system

**Figure 6:**
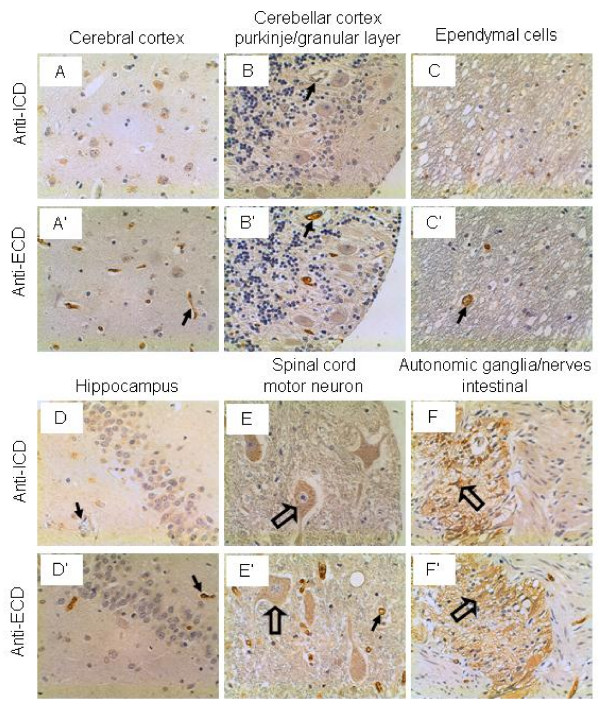
**IL-17RC expression in the nervous system.** Arrows: endothelium (capillaries in A', D', and E'; and arterioles in B, B', C', and D); and open arrows: motor neurons (E and E') and autonomic ganglia & nerves (F and F'). Original magnification: × 400.

The neurons in the cerebral and cerebellar cortices were not stained by either the anti-ICD or anti-ECD antibodies (Figure 6,A/A' and 6B/B'). Neither the ependymal cells nor the neurons in the hippocampus were stained by either antibodies (Figure 6,C/C' and 6D/D'). Of note, the vascular endothelium in the aforementioned tissues was not stained by the anti-ICD antibodies but stained strongly by the anti-ECD antibodies. It has been demonstrated that IL-17A plays an important role in the experimental autoimmune encephalomyelitis [26,48-57]. Yet, it is not clear whether IL-17RC in the vascular endothelium of the brain plays any role in this autoimmune disease. Interestingly, the motor neurons in the spinal cord and the intestinal autonomic ganglia were stained weakly by both antibodies (Figure 6,E/E' and 6F/F').

### Liver, gallbladder, pancreas, and salivary gland

**Figure 7:**
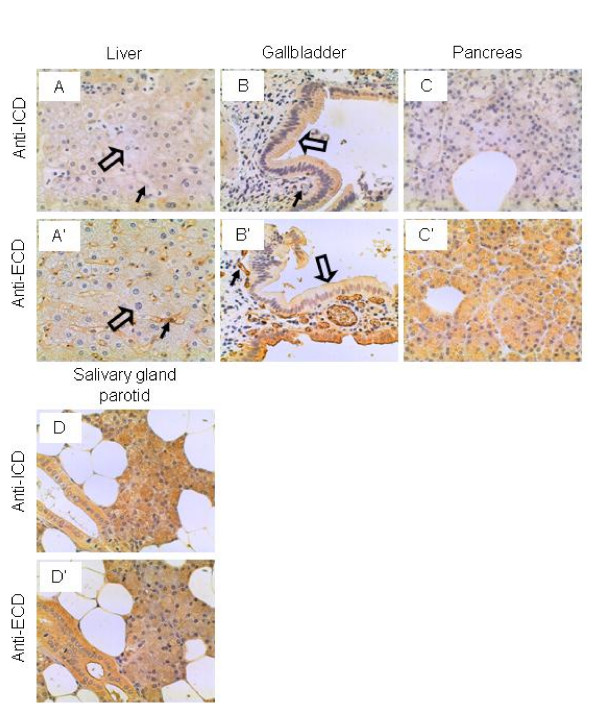
**IL-17RC expression in the liver, gallbladder, pancreas, and salivary gland.** Arrows: endothelium; and open arrows: hepatocytes (A and A') and epithelium (B and B'). Original magnification: × 400.

The hepatocytes were not stained by either the anti-ICD or anti-ECD antibodies, although the sinusoid endothelium was stained intermediately by the anti-ECD but not the anti-ICD antibodies (Figure 7,A and 7,A'). The gallbladder epithelium was stained weakly and intermediately by the anti-ICD and anti-ECD antibodies, respectively (Figure 7,B and 7,B'). The glandular cells of the pancreas were not stained by the anti-ICD antibodies, whereas they were stained intermediately by the anti-ECD antibodies (Figure 7,C and 7,C'). It has been reported that IL-17A in combination with IL-1 and TNFα induced IL-6 secretion by the pancreatic periacinar myofibroblasts, which might play an important role in the pathophysiology of acute pancreatitis [58]. Whether the pancreatic glandular cells respond to IL-17A is yet to be determined. Interestingly, the glandular cells of the parotid salivary gland were stained strongly by both antibodies (Figure 7,D and 7,D').

### Urinary and respiratory systems

**Figure 8:**
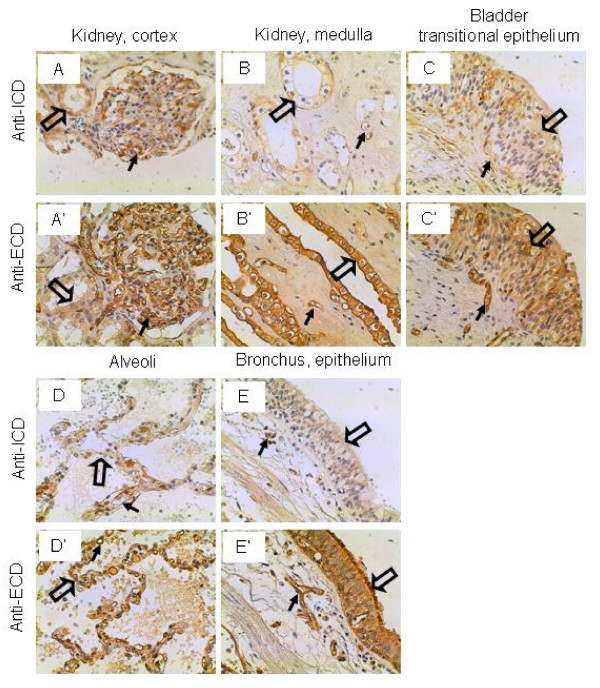
**IL-17RC expression in the urinary and respiratory systems.** Arrows: endothelium; and open arrows: epithelium (A, A', B, B', C, C', E, and E') and alveolar lining cells (D and D'). Original magnification: × 400.

The glomerulus of the renal cortex was stained strongly by both the anti-ICD and anti-ECD antibodies (Figure 8,A and 8,A'), due possibly to the vascular nature of the glomerular tufts. In the renal medulla, the tubular epithelium was stained weakly and strongly by the anti-ICD and anti-ECD antibodies, respectively (Figure 8,B and 8,B'). Similarly, the transitional epithelium of the bladder was stained weakly and strongly by the anti-ICD and anti-ECD antibodies, respectively (Figure 8,C and 8,C'). In the lung, the alveolar lining cells and the capillary endothelial cells were stained weakly and intermediately by the anti-ICD and anti-ECD antibodies, respectively (Figure 8,D and 8,D'). However, the bronchial epithelium was not stained by the anti-ICD antibodies but stained strongly by the anti-ECD antibodies (Figure 8,E and 8,E'). IL-17A and IL-17F have been shown to play an important role in airway inflammation [31,32,57]. IL-17RA is localized to basal airway cells in human lung tissue and functional IL-17RA signaling occurs on the basolateral surface of human bronchial epithelial cells [59]. In comparison, IL-17RC as recognized by the anti-ECD antibodies appeared to be expressed by all cell types in the bronchial epithelium and the underneath vascular endothelial cells (Figure 8,E'). It is not clear what the role is for IL-17RC in these tissues.

### Skin and lymphatic tissues

**Figure 9:**
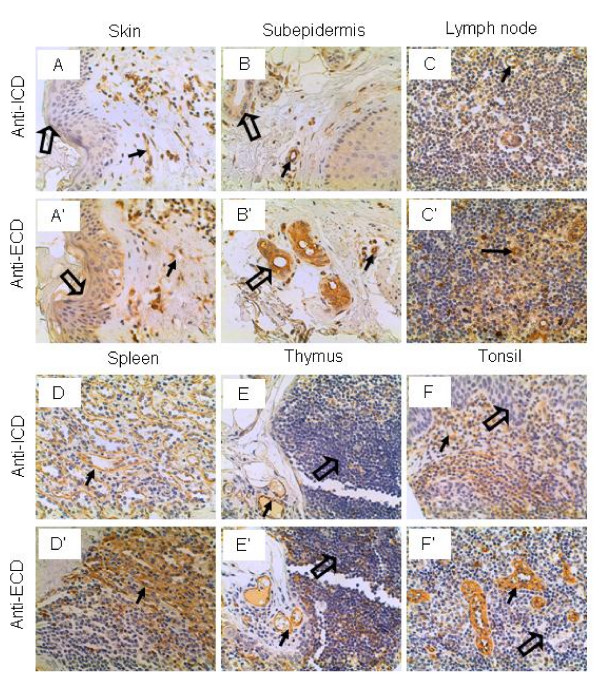
**IL-17RC expression in the skin and lymphatic tissues.** Arrows: endothelium; and open arrows: squamous epithelium (A and A'), glandular epithelium (B and B'), and lymphocytes (E, E', F, and F'). Original magnification: × 400.

The squamous epithelium in the skin were not stained by the anti-ICD antibodies but stained intermediately by the anti-ECD antibodies (Figure 9,A and 9,A'). In the subepidermis, the glandular cells (of either the sebaceous or sweat glands) were stained weakly and strongly by the anti-ICD and anti-ECD antibodies, respectively (Figure 9,B and 9,B'). The lymphatic cells in the lymph node, spleen, thymus and tonsil were not stained by either antibodies, although the lymphatic sinusoid or vascular endothelium was stained positively by both antibodies (Figure 9,C/C',D/D',E/E', and 9F/F'). This observation is consistent to the previous report that IL-17RC mRNA levels were very low in the thymus and spleen [35].

### Miscellaneous tissues

**Figure 10:**
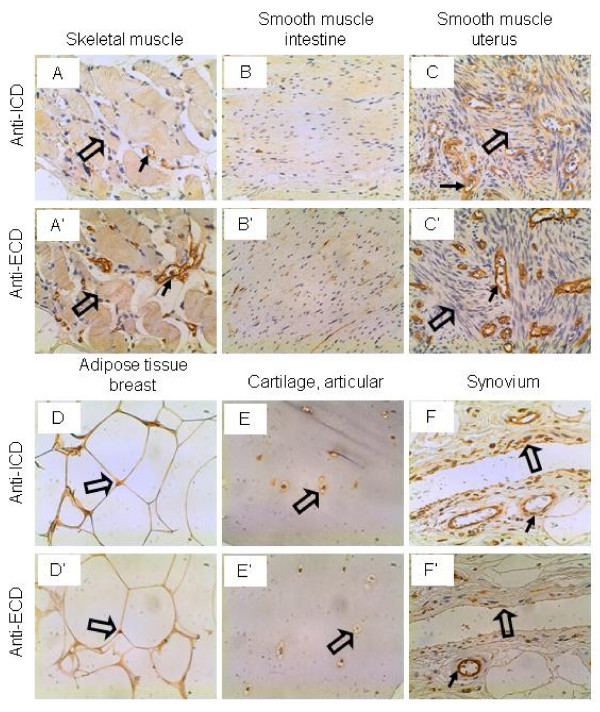
**IL-17RC expression in miscellaneous tissues.** Arrows: endothelium; and open arrows: skeletal muscle cells (A and A'), smooth muscle cells (C and C'), adipocytes (D and D'), articular chondrocytes (E and E'), and synovial cells (F and F'). Original magnification: × 400.

The skeletal muscle cells were not stained by the anti-ICD antibodies but stained weakly by the anti-ECD antibodies, although the vascular endothelium was stained strongly by both antibodies (Figure 10,A and 10,A'). The smooth muscle cells of the intestine and uterus were not stained by either antibodies (Figure 10,B/B' and 10C/C'). However, the vascular endothelium in uterus was stained strongly by both antibodies (Figure 10,C/C'). The adipocytes from the breast tissue were stained weakly by both antibodies (Figure 10,D and 10,D'). In articular cartilage, the chondrocytes were stained weakly by the anti-ICD antibodies but not stained by the anti-ECD antibodies (Figure 10,E and 10,E'). Similarly, the synovial lining cells in the joint were stained intermediately by the anti-ICD antibodies but not stained by the anti-ECD antibodies (Figure 10,F and 10,F'), even though the vascular endothelium was stained strongly by both antibodies. It is not known how IL-17RC contributes to the pathogenesis of rheumatoid arthritis [21,22].

Previous studies have shown that *IL-17RC *gene encodes different mRNA splice isoforms and different proteins [35,39,45,46]. The present study systematically examined the IL-17RC protein expression in 53 human tissues. The results showed different IL-17RC proteins were expressed differentially in different human tissues, based on the signals detected by two distinct antibodies. The anti-ECD antibodies recognized several endogenous IL-17RC protein bands that were larger than the recombinant full-length IL-17RC in a Western blot analysis, leading to a speculation that the anti-ECD antibodies recognized the glycosylated IL-17RC protein [46]. This is likely due to the fact that the immunogen used to generate the antibodies was expressed in mammalian cells and thus probably glycosylated. On the other hand, the anti-ICD antibodies were generated by using the 14-amino acid peptide, which possibly had higher affinity for non-glycosylated IL-17RC protein. The true identity of the stained IL-17RC proteins needs to be further investigated. Nevertheless, it is unlikely that the two antibodies recognized some proteins other than IL-17RC, because it has been shown that both antibodies recognized the recombinant full-length IL-17RC and had no cross-reactivity with IL-17RA or IL-17RB – two homologues of IL-17RC [46].

It is known that IL-17RC shares about 20% homology to other IL-17 receptor family members [2]. The tissue-specific expression of other IL-17 receptor family members has been investigated mainly through Northern blot and/or RT-PCR analysis of mRNA expression. For example, IL-17RA has been shown to be ubiquitously expressed in many cell lines and tissues [5,60]. Expression of IL-17RB is most pronounced in fetal and adult liver, kidney, pancreas, testis, brain, colon, and small intestines, but is absent in peripheral blood leukocytes and lymphoid organs [7,8]. IL-17RD is also named Similar expression to fgf genes (Sef), which has several isoforms that are differentially expressed in different tissues. Human Sef-a transcript was highly expressed in both fetal and adult brain, pituitary, tonsils, spleen, adenoids, fetal kidney, liver, testes, and ovary, and moderate levels were detected in primary aortic endothelial cells, human umbilical vein endothelial cells, and adrenal medulla. Low levels of Sef-a were observed in adrenal cortex, barely detected in placenta, and absent in thyroid. In contrast, Sef-b transcript was highly expressed in thyroid and testes and moderately expressed in pituitary, fetal brain, and human umbilical vein endothelial cells; the remaining tissues were either negative or expressed at barely detectable levels [61]. Human IL-17RE has not been characterized, although mouse IL-17RE mRNAs have been shown to be mainly expressed in the lung, kidney, stomach, and testis, but not in heart, liver, spleen, or brain [62]. It would be interesting to compare the protein expression profiles of all IL-17 receptor family members in future studies.

## Conclusion

By immunohistochemical staining, IL-17RC protein was detected in most human tissues. The cell types that expressed IL-17RC protein include the myocardial cells, vascular and lymphatic endothelial cells, glandular cells (of the adrenal, parathyroid, pituitary, thyroid, pancreas, parotid salivary, and subepidermal glands), epithelial cells (of the esophagus, stomach, intestine, anus, renal tubule, breast, cervix, Fallopian tube, epididymis, seminal vesicle, prostate, gallbladder, bronchus, lung, and skin), oocytes in the ovary, Sertoli cells in the testis, motor neurons in the spinal cord, autonomic ganglia in the intestine, skeletal muscle cells, adipocytes, articular chondrocytes, and synovial cells. In particular, IL-17RC protein expression was very high in most vascular and lymphatic endothelium and squamous epithelium. The epithelial cells of the breast, cervix, Fallopian tube, kidney, bladder and bronchus also expressed high levels of IL-17RC, so did the glandular cells in the adrenal cortex, parotid salivary and subepidermal glands. In contrast, IL-17RC protein was not detectable in some cell types such as the smooth muscle cells, fibroblasts, antral mucosa of the stomach, mucosa of the colon, endometrium of the uterus, neurons of the brain, hepatocytes, or lymphocytes. Nevertheless, IL-17RC was expressed in the vascular endothelium within the tissues where the IL-17RC-negative cells resided. It is unlikely that the negative staining was due to a technical artifact (false negative), because all tissue cores were mounted and simultaneously stained on a single slide and the positive tissue cores or positive vascular endothelium within the negative tissue cores served as internal positive controls. Therefore, the lack of IL-17RC expression in some of the tissues reflects the tissue-specific pattern of gene expression, which implies that IL-17RC has no functional role in these tissues or the particular cell types.

It is speculated that IL-17RC may play a role in almost all human tissues. It is further envisioned that different IL-17RC proteins may determine the distinct cellular responses to the ligands, particularly in those cell types that do not express the IL-17RC protein as recognized by the anti-ICD antibodies (for example, the squamous epithelium of the esophagus, anus, and skin; and the epithelium of breast, cervix, Fallopian tube, prostate, and bronchus) but do express high levels of the IL-17RC protein as recognized by the anti-ECD antibodies.

The ligands of IL-17RC have been identified as IL-17A and IL-17F and the intracellular signaling pathway for IL-17RA has been revealed [45,48]. The IL-17 cytokines have been shown to require a heterodimer of IL-17RA and IL-17RC for mediating their signals [42,47]. However, the signaling pathway downstream to IL-17RC is still a mystery [14]. Nevertheless, in those tissues wherein IL-17RC is expressed, IL-17RC may participate in inflammatory and immune responses that have been demonstrated in studies of IL-17A and IL-17F [14,57,63], although the direct role of IL-17RC is yet to be determined. The present findings of IL-17RC expression in most human tissues may stimulate further investigation into this under-studied but presumably important member of IL-17 receptor family.

## Methods

### Antibodies and Reagents

Rabbit anti-IL-17RC intracellular domain antibodies (anti-ICD) that recognize an intracellular domain (DSYFHPPGTPAPGR) of IL-17RC protein were affinity-purified [35,39,40,46]. Goat anti-IL-17RC extracellular domain antibodies (anti-ECD) were generated using the extracellular domain of human IL-17RC isoform #3 (exon 7 was spliced out) as immunogen (Catalog Number AF2269, R&D Systems Inc., Minneapolis, MN) [46,47]. Due to the polyclonal nature, the epitopes recognized by the anti-ECD antibodies were unknown, which might be any exons from 1 to 16 except exon 7. VECTSTAIN elite ABC Reagent and DAB Substrate Kit were from Vector Laboratories, Burlingame, CA. The human normal tissue microarray (TMA) slides (Version CHTN2002N1) were provided by the Cooperative Human Tissue Network (CHTN). All tissue cores were made from the formalin fixed paraffin embedded human normal adult tissues, except the parathyroid and lymphatics that were from the benign parathyroid adenoma and lymphangioma, respectively (Table 1). Each tissue type was represented by four 0.6-mm tissue cores with a thickness of 4-μm on each slide. The tissue type and quality of TMA slides were assured by the provider's pathologist. The human tissue samples have been de-identified in such a way that only the age and sex of each donor were available to the investigators.

### Immunohistochemical staining

The TMA slides were deparaffinized and rehydrated. Antigen retrieval was performed by high-temperature (microwave) incubation in 0.01 mol/L of citric acid buffer (pH 6.0) for 3 times of 4 min each. The slides were cooled down to room temperature in about 10 min in citric acid buffer and then transferred to phosphate-buffered saline (pH 7.4). Endogenous peroxidase was blocked in a solution of 3% hydrogen peroxide in methanol for 30 min. The slides were incubated for 20 min in a humidified chamber in 10% normal goat or rabbit serum (Vector Laboratories) and then in primary antibody solution overnight at 4°C. Positive and negative control slides were the prostate tumor slides with or without IL-17RC expression as determined previously [40,46] and were run with or without primary antibodies. Primary antibodies were used at 7.5 μg/ml for the anti-ICD antibodies and 0.25 μg/ml for the anti-ECD antibodies. The VECTSTAIN elite ABC Reagent and DAB Substrate Kit were used according to the manufacturer's protocol [40,64,65]. The slides were counterstained in Mayer's hematoxylin. The staining conditions were optimized by using several test slides. Similar results were obtained from two TMA slides for each primary antibody. The stained slides were assessed independently by all authors and a consensus was reached. In order to present relative levels of IL-17RC expression, the intensity of IL-17RC staining for a particular cell type in the tissues was graded in this way: - = no staining; + = weak; ++ = intermediate; +++ = strong.

## List of abbreviations

anti-ECD: anti-extracellular domain; anti-ICD: anti-intracellular domain; ERK: extracellular signal-regulated kinase; FGF: fibroblast growth factor; IL-17: interleukin-17; IL-17R: interleukin-17 receptor; TMA: tissue microarray; TNF: tumor necrosis factor.

## Competing interests

The authors declare that they have no competing interests.

## Authors' contributions

DG analyzed the data and critically reviewed the manuscript for intellectual content; ZY conceived the design, collected and analyzed the data, and drafted the manuscript. All authors read and approved the final manuscript.
